# Cardiovascular Disease Chemogenomics Knowledgebase-guided Target Identification and Drug Synergy Mechanism Study of an Herbal Formula

**DOI:** 10.1038/srep33963

**Published:** 2016-09-28

**Authors:** Hai Zhang, Shifan Ma, Zhiwei Feng, Dongyao Wang, Chengjian Li, Yan Cao, Xiaofei Chen, Aijun Liu, Zhenyu Zhu, Junping Zhang, Guoqing Zhang, Yifeng Chai, Lirong Wang, Xiang-Qun Xie

**Affiliations:** 1 College of pharmacy, Second Military Medical University; Department of Pharmacy, Third Affiliated Hospital of Second Military Medical University, Shanghai 200433, China; 2 Department of Pharmaceutical Sciences and Computational Chemical Genomics Screening Center, School of Pharmacy; National Center of Excellence for Computational Drug Abuse Research; Drug Discovery Institute; Departments of Computational Biology and Structural Biology, School of Medicine, University of Pittsburgh, Pittsburgh, Pennsylvania 15260, United States

## Abstract

Combination therapy is a popular treatment for various diseases in the clinic. Among the successful cases, Traditional Chinese Medicinal (TCM) formulae can achieve synergistic effects in therapeutics and antagonistic effects in toxicity. However, characterizing the underlying molecular synergisms for the combination of drugs remains a challenging task due to high experimental expenses and complication of multicomponent herbal medicines. To understand the rationale of combination therapy, we investigated Sini Decoction, a well-known TCM consisting of three herbs, as a model. We applied our established diseases-specific chemogenomics databases and our systems pharmacology approach TargetHunter to explore synergistic mechanisms of Sini Decoction in the treatment of cardiovascular diseases. (1) We constructed a cardiovascular diseases-specific chemogenomics database, including drugs, target proteins, chemicals, and associated pathways. (2) Using our implemented chemoinformatics tools, we mapped out the interaction networks between active ingredients of Sini Decoction and their targets. (3) We also *in silico* predicted and experimentally confirmed that the side effects can be alleviated by the combination of the components. Overall, our results demonstrated that our cardiovascular disease-specific database was successfully applied for systems pharmacology analysis of a complicated herbal formula in predicting molecular synergetic mechanisms, and led to better understanding of a combinational therapy.

In order to enhance efficacy and reduce toxicity simultaneously, combinational drug therapy is becoming a popular strategy for many disease treatments in clinics, such as HIV cocktails for AIDS treatment. In fact, traditional Chinese medicine (TCM) formulae are real-life clinical-tried medicinal herbal combination therapies that have been used for millennia to clinically treat human beings. For complicated or multi-factorial diseases, emerging evidence indicates that using multiple drugs that have common or different pharmacological targets often yields better therapeutic efficacy than the use of a single medication^1^,^2^. Typically, an herbal medication formula consists of several natural herbs and each of them performs its own function, i.e., principal (Jun), assistant (Chen), complement (Zuo) and guide (Shi) components based on their roles in the prescription. Logically, the principal herb possesses the main pharmacological actions, and the others serve synergistic actions to get maximal therapeutic efficacy with minimal adverse effects^3^.

Multi-herbal formulae exert their therapeutic efficacies through the synergistic effects of multiple ingredients on multiple targets^4^,^5^,^6^. It is vital to reveal the targets of active components in order to understand the systematic mechanism of herbal formulae. Sini Decoction (SND), an ancient TCM formulation, consisting of three different herbs: *Aconitum carmichaelii*, *Zingiber officinale*, and *Glycyrrhiza uralensis,* has been used for hundred years in Chinese practical clinics. It was officially recorded in Chinese pharmacopoeia and has been used to treat cardiovascular disease for many years. SND has been applied as a life-saving drug to treat patients with heart failure (HF), myocardial infarction (MI), shock and other serious diseases^7^,^8^,^9^. According to the literature, SND can ameliorate lipid profile, improve microcirculation^10^ and regulate blood pressure to help blood reflux to the heart and flux rapidly into the end of circulation. This can keep the patients warm and help to treat rheumatism, general debility, cardiac weakness, weak circulation and decreased kidney function^11^. According to the Figure S1, the targets involved in SND indications, such as coronary disease, myocardial infarction, shock and heart failure, overlap with each other and also overlap with the targets involved in other cardiovascular diseases. In SND, *Aconitum carmichaelii*, the principal herb, *Zingiber officinale*, an assistant herb, and *Glycyrrhiza uralensis*, as a complement and guide herb, work together to get maximal therapeutic efficacy with minimal adverse effects^12^. The difficulty of isolating the natural chemical ingredients and the high cost of bioassay approaches to identify the potential targets of essential ingredients hampered the characterization and target identification of natural herbal medicines and limited our understanding of combinational therapies^13^.

With modern analytical technologies, a large amount of natural chemical structure data has been processed and has become available, laying a foundation for further analysis. The application of computational methods combined with network pharmacology is an indispensable and powerful way to evaluate pharmacological effects at the molecular level for TCM and to analyze the complex interactions between several small molecules and multiple target proteins in biological systems^5^,^14^,^15^. Existing experimental techniques to detect polypharmacology have their limitations. First, with techniques such as the so-called target-fishing approach, the main drawback is that it cannot rule out the influence of nonspecific binding. Second, it is often tedious, time-consuming, costly and complicated to screen and analyze large data sets from natural medicinal products. Therefore, the application of established virtual-screening techniques can be successfully adopted for network pharmacological analysis^16^,^17^. Since network pharmacology is highly interconnected with chemogenomics, which describes the target-ligand structure activity relationship^13^, a unique computational tools that deal with comprehensive chemogenomics information are needed^18^,^19^,^20^. Therefore, use of network pharmacology tools is needed to construct the interaction network between active ingredients and targets; Targets and pharmacological actions can then be verified in the model.

There are many reported computational approaches that have been applied for target modeling and prediction. Among these, Alzheimer’s disease-specific chemogenomics database has been constructed for target identification and network pharmacology analysis of the chemical ingredients from seed extract of Platycladus orientalis^21^. The technologies have also been applied in drug abuse polypharmacology research^18^, as well as molecular mechanism studies of Fufang Xueshuantong Capsule^18^,^22^. These platforms were integrated with our established algorithms and programs: GPU-accelerated machine learning algorithms for ligand specificity and function predictions^23^,^24^,^25^,^26^,^27^, molecular fingerprint-based TargetHunter© program for drug target identification^28^, and receptor homology modeling and virtual screening approaches for ligand screening of cannabinoid receptor^29^,^30^. All of these techniques have been demonstrated to be effective and efficient, laying the foundation for virtual screening, target identification, and network systems pharmacology studies^18^,^22^,^31^.

The present studies have been also based on our experimental data. Our previous studies have identified the chemical ingredients in SND, in which 51 peaks were detected^32^,^33^,^34^,^35^,^36^,^37^. The results provided a chemical material basis for further network pharmacological analysis in the present studies. Previous studies also revealed the role of absorption, distribution, metabolism and excretion of the three herbs, as well as their synergistic effects in SND^38^,^39^,^40^. In the present study, we constructed a cardiovascular-diseases (CVD) specific database, with an integrated chemogenomics knowledgebase consisting of chemicals, genes/protein targets, signaling pathways information and our established chemoinformatics tools^18^,^22^,^28^. Subsequently, we mapped out a biological network of interactions between chemical compounds and target proteins at the molecular level, and then investigated the potential molecular synergisms and antagonisms, followed by verification of the predicted targets through carrying out protein binding experiments *in vitro*. Finally, we validated synergetic pharmacological effects of SND on modulating heart failure in a coronary artery ligation rat model. The combined approaches offer a deep understanding of pharmacological mechanisms of combination therapy for herbal medication formulae by using SND as an illustration. It is a novel and efficient way to clarify the compatibility mechanisms of TCM formulea. Taken together, the constructed CVD-specific database and established systems pharmacology network analysis approaches reported here provide significant new insight the pharmacological mechanisms of multi-ingredient natural products. The derived rationale will have a valuable impact on combinational therapy treatment of CVD in clinics.

## Results

### CVDPlatform Construction and Validation

*CVDPlatform* (www.cbligand.org/CVD) was constructed from 984 achieved target proteins related to cardiovascular diseases, 924 CVD drugs that have been either FDA-approved or are in clinical trials, 2080 active chemical compounds associated with therapeutic targets of CVDs, 276 cardiovascular-related pathways, and 350,765 references. Since some important targets of cardiovascular diseases have no available human crystal structures, their homology models were built and added into our database. For example, the homology model of a human beta-1 adrenergic receptor (ADRB1) was built according to the crystal structure of turkey beta-1 adrenergic receptor (PDB Entry: 2Y00) co-crystal structure^41^.

**Figure 1:**
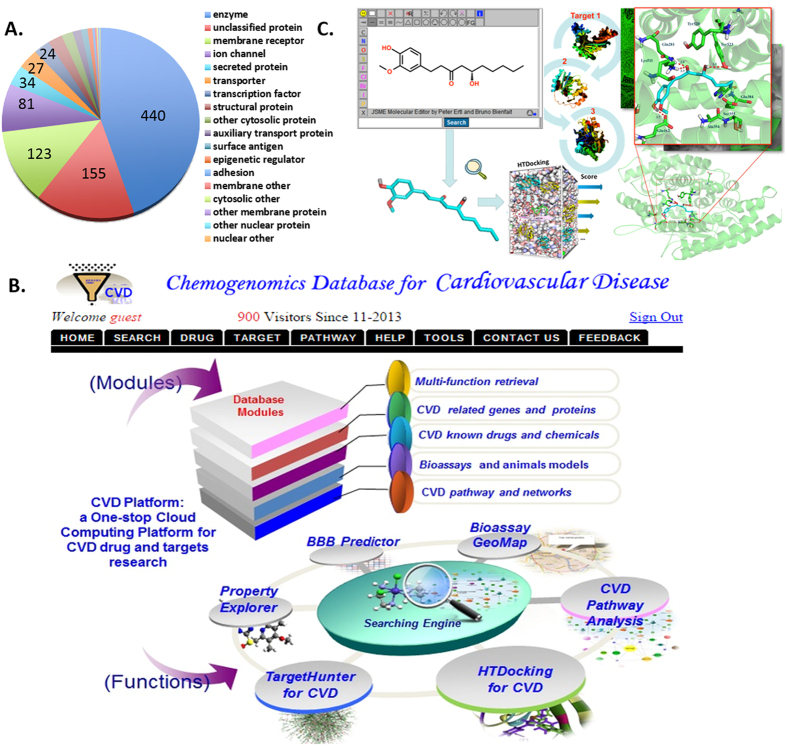
Overview of Targets in CVDPlatform and HTDocking for system pharmacology analysis. (**A**) Cardiovascular disease targets’ classification. (**B**) Website interface for CVDPlatform. (**C**) HTDocking procedure. We used JSME as a molecule editor implement in our database^70^.

**Figure 2:**
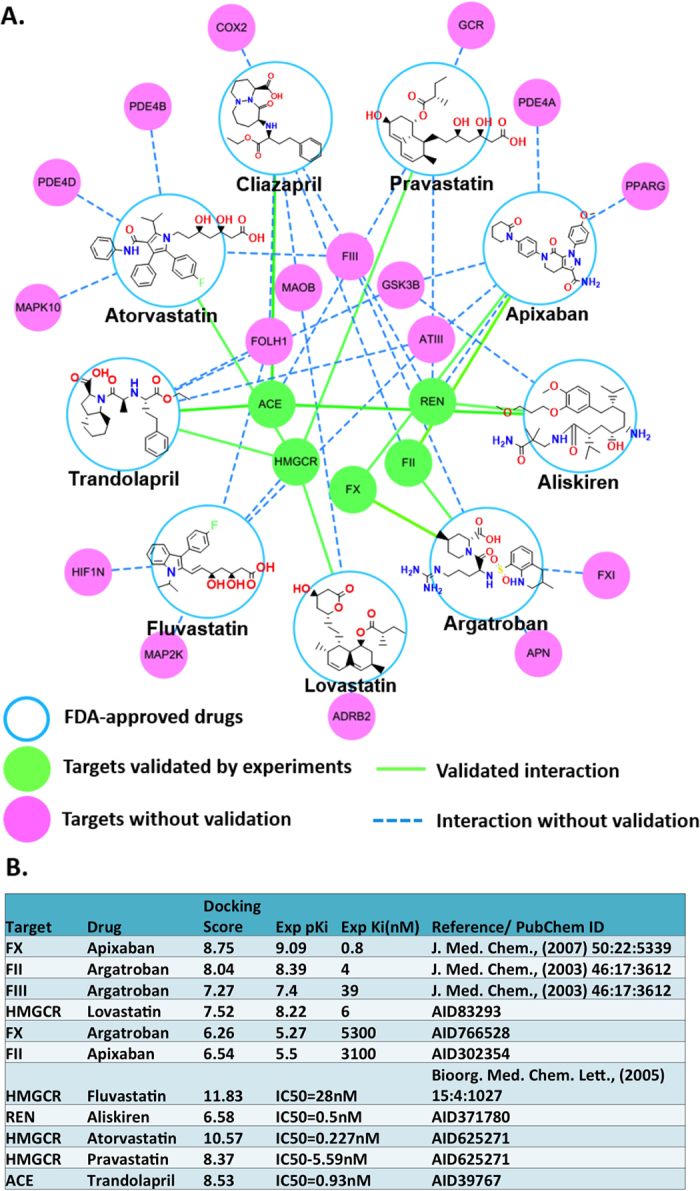
Application of HTDocking program in FDA-approved CVD drugs. (**A**) Interaction network between approved CVD drugs and their potential targets. Large circles with chemical structures represent drugs, and nodes stand for targets. Green nodes are targets validated by experiments, and pink nodes are predicted targets without validation. The edges stand for the interactions: blue dashed edges stand for the interactions without validation; green solid edges stand for the validated interactions. (**B**) Comparison between the docking score and bioassays data.

Figure 1A illustrates the targets in the cardiovascular diseases database (CVDPlatform), including 440 enzymes, 123 membrane receptors and 81 ionic channels, *etc.* As a validation procedure, the in-house chemoinformatic tool HTDocking in the CVD database was used to predict potential targets for nine FDA-approved drugs, including four 3-hydroxy-3-methylglutaryl-coenzyme A reductase (HMGCR) inhibitors (Atorvastatin, Fluvastatin, Pravastatin and Lovastatin), two angiotensin converting enzyme (ACE) inhibitors (Cilazapril and Trandolapril), a renin (REN) inhibitor (Aliskiren), a prothrombin (FII) antagonist (Argatroban) and a coagulation factor X (FX) inhibitor (Apixaban). The website interface was shown in Fig. 1B. The procedures of HTDocking were shown in Fig. 1C. Potential target proteins of these 9 anti-CVD drugs were predicted and ranked by docking scores, and the interaction network was shown in Fig. 2A. The results showed that most of the known therapeutic targets of these 9 drugs ranked highly. For instance, HMGCR ranked first in the target proteins lists of Pravastatin, Lovastatin and Fluvastatin. The predicted binding affinities (via docking scores expressed as -log_10_K_d_) for these targets were also consistent with the bioactivity data (Fig. 2B). Furthermore, some additional predicted interactions had been validated by bioassays reported in literature or PubChem, indicating the reliability of the HTDocking program in CVDPlatform (Table S1). Statistical analysis was performed on the number of drugs in the different development phases according to their therapeutic targets. The corresponding targets were ranked according to the total number of drugs, and the top 20 targets were listed in Figure S2. In addition, similar targets were emphasized for the cardiovascular diseases as well as for the specific indications of SND, such as HF and MI (as shown in Figure S3), indicating the reliability of using the cardiovascular diseases database to inspect SND. The statistical analysis of CVD targets was also plotted out according to the pathways involved with the CVD targets (Figure S4).

### Target Prediction and Network Pharmacological Analysis

Thirty-three targets related to the therapeutic effect of SND were mapped out with 31 components by molecular docking using CVDPlatform. Among them, interactions validated by others can be easily found among our predicted results through literature data mining, as shown in Table S2. Other 9 components (deoxyaconitine (4), neoline (15), talatisamine (16), 14-acetyl-talatisamine (18), talatizidine (19), coryneine (23), Glycyrrhizic Acid (26), isoliquiritigenin (31), and glycyrrhetic acid (36)) were ruled out due to the low docking score (<5.0) to all the candidate proteins. Most candidate proteins were shared by more than one component, including ADRB1, ADRB2, M3AChR (M3 muscarinic acetylcholine receptor), FII, HMGCR, ACE and REN. These potential targets might play critical roles in achieving their therapeutic effects. Therefore, a component-target interaction network for SND was generated to investigate the therapeutic, synergistic and antagonistic effects of these components. As illustrated in Figure S2A, the bilateral graphical network incorporated 64 nodes and 178 edges, where the edges encoded interaction and the nodes represented potential targets (in squares) or compounds (in circles). The outside target proteins with small sizes were only targeted by one component. The inside potential targets (in squares) were linked to multiple compounds^18^.

**Figure 3:**
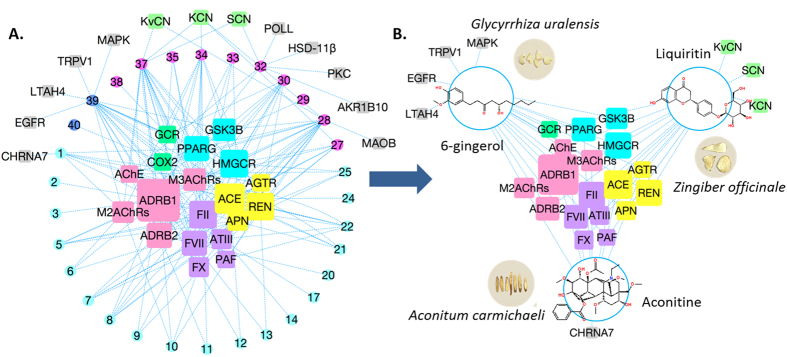
Network pharmacology analysis for components in Sini Decoction. (**A**) Predicted compound-target interactions between 33 major components in SND and CVD targets. (**B**) Predicted interaction network between the three selected constituents (aconitine, liquiritin and 6-gingerol) and CVD targets. Squares represent targets, and circles represent compounds. Different colors are used to distinguish targets from different physiological systems and constituents from different herbs: (Outer ring) Constituents from *Aconitum carmichaeli* are colored in cyan, from *Glycyrrhiza uralensis* are magenta colored and from *Zingiber officinale* are colored in blue; (Inner squares) Targets in autonomic nervous system are colored in pink, in coagulation pathway are colored in purple, and in renin-angiotensin-aldosterone system are colored in yellow. In addition, the sizes of the squares refer to the number of interactions. The edges stand for the interactions: blue dashed edges stand for the interactions without validation; green solid edges stand for the validated interactions.

Construction of a component-target interaction network for a specific herbal formula is reasonable and helpful to identify potential targets and to further evaluate synergistic effects in the context of disease pathways^42^. By using the network pharmacology approach to further investigate the target proteins connected with multiple components, we found that most of the shared targets belong to a few physiological systems, such as the renin-angiotensin-aldosterone system (RAAS), the coagulation pathway, lipid metabolism and the autonomic nervous system (Fig. 3A).

### Pharmacological Evaluation and Molecular Compatibility Mechanism Study of Key Ingredients in SND

According to the results of the component-target interaction and pharmacological evaluation of SND, aconitine, 6-gingerol and liquiritin were selected to construct an interaction network of ingredient-target, as shown in Fig. 3B. When comparing Fig. 3B with Fig. 3A, it was found that the targets of these three selected ingredients covered almost all targets of 31 components in SND. As such, aconitine, 6-gingerol and liquiritin were selected as the representative ingredients in aconitum, ginger and licorice to clarify the molecular compatibility mechanism of SND. Additional reasons can be found in the following.

The main active components in *Aconitum carmichaelii* are alkaloids, including diester-diterpene alkaloids (DDAs), monoester-diterpene alkaloids (MDAs), and amine-diterpenoid alkaloids (ADAs). The three forms of alkaloids were progressively transformed from DDAs to MDAs and finally ADAs during the heating process. It was reported that the cardiac functions of DDAs were much stronger than MDAs and ADAs, but DDAs were the most toxic alkaloids in *Aconitum carmichaelii*. Aconitine, one of the DDAs, had potential targets closely related to cardiovascular actions of *Aconitum carmichaelii*, involving ADRB1, ADRB2, Acetylcholinesterase^43^, ACE and HMGCR, according to our virtual docking results. In addition, aconitine has been studied for many years and its pharmacological effects are clear. As such, aconitine was selected as the representative ingredient in *Aconitum carmichaelii*.

The major active components of *Zingiber officinale* are gingerols. 6-Gingerol has the highest content among all gingerols in *Zingiber officinale.* Up to now, more than 100 papers have been reported with regard to the pharmacological effects of 6-ginerol. Moreover, according to the results of virtual docking, the potential targets of 6-gingerol such as HMGCR covered important predicted therapeutic targets for ginger. Thus 6-gingerol was selected as the representative ingredient in *Zingiber officinale*.

Flavonoids and saponins are the main components in *Glycyrrhiza uralensis*. Saponins would be transformed into flavonoids under the intestinal flora *in vivo*, and the carbohydrate chains of saponins are usually metabolized and cut off from the steroids during the metabolism process. So flavonoids were selected as the main components for virtual docking. According to the results of virtual docking, the targets of liquiritin represented most of the targets for liquorice in SND, including ion channels and ACE, *et*c. Therefore liquiritin was selected as the representative ingredient in *Glycyrrhiza uralensis*.

### Biological Evaluation of Pharmacological Actions of Combined Three Ingredients and Component Extracts of SND

The component extracts of three herbs were extracted and prepared, and their compatibility ratio was designed according to the contents of diester-diterpenoid alkaloids of *Aconitum carmichaelii*, total flavonoids and saponins in *Glycyrrhiza uralensis*, and total gingerols of *Zingiber officinale* in Sini Decoction. The standards of aconitine, liquiritin and 6-gingerol were obtained from China’s National Institute for the Control of Pharmaceutical and Biological Products (Beijing, China), and their purity was more than 98%. Their compatibility ratio was performed according to their contents in SND, which was 1:7:140.

**Figure 4:**
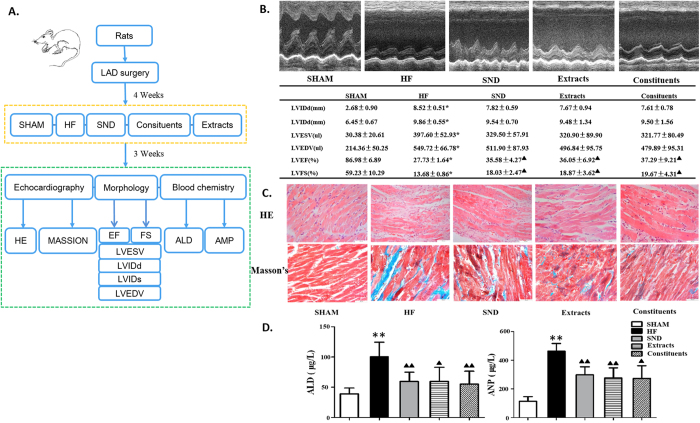
Effect of SND, herb extracts, and the combination of three ingredients on heart function in rat model of heart failure. (**A**) Experimental procedures. (**B**) Echocardiography and data in rats with sham operation, HF, and HF with different drug treatments. (**C**) MASSON and HE stained slides (histology) of the left ventricular tissue from above rat groups. *p < 0.05 comparing HF and sham animals, ^▲^p < 0.05 comparing drug treatment and HF animals, **p < 0.01 comparing HF and sham animals, ^▲▲^p < 0.01 comparing drug treatment and HF animal. (**D**) Effect of drug treatment on the plasma concentration of aldosterone (ALD) and amino peptide N (APN).

As shown in Fig. 4A–D, the pharmacological actions were evaluated by echocardiographic assay, morphology and biological chemistry. As shown in Fig. 4B, the rat heart failure (HF) models show a significant increase in the diameters in systole and diastole (LVIDs or LVIDd), left ventricular volume (LVESV or LVEDV), followed by the reduced left ventricular ejection fraction (LVEF) and left ventricular fractional shortening (LVFS) compared to the shaw operation group as an indicator for an impaired heart function. After three weeks of drug treatment, echocardiography showed a significant decrease in the diameters and volumes, but an increased in LVEF and LVFS in the drug treatment group versus the model group (*P* < 0.05), indicating an improved heart function after treatment. The drug treatment groups also showed increased LVIDs, LVIDd, LVESV and LVEDV versus the sham group (*P* > 0.05), but not significantly. Moreover, as shown in Fig. 4C, the hematoxylin and eosin (HE) and MASSON-stained images of left ventricular tissue showed that cardiomyocytes in the sham group were orderly arranged, and the nuclei were lightly stained. Thickening and lengthening of myocardial fibers could be observed in the model groups. Cardiomyocyte hypertrophy and cellular degeneration improved in the different drug groups in contrast with those in the model group. Furthermore, as shown in Fig. 4D, the concentration of ALD and ANP significantly increased in HF model group compared with sham group (*P* < 0.01), but were significantly decreased in drug therapeutic group compared with HF model group (*P* < 0.05), suggesting that the RAAS hyperthyroidism in HF rat was inhibited through the drugs treatment. Taken together, these results showed that the HF model was successfully established and that treatment with SND, component extracts, or 3-ingredient combination each results in a significant improvement in heart function in HF rats.

### Experimental Validation of Key Representative Ingredients binding to Beta-1 Adrenergic Receptor

ADRB1 was the potential target linked to the largest number of components in our prediction. ADRB1 can increase heart rate in the sino atrial (SA) node, and elevate contractility and automaticity of the ventricular cardiac muscle, thus increasing cardiac output^44^. Both the molecular docking and pharmacological evaluation results showed that propranolol, a well-known beta-adrenergic antagonist, can inhibit the cardiac function induced by aconitine, suggesting that aconitine acts on ADRB1 to achieve its cardiac function.

**Figure 5:**
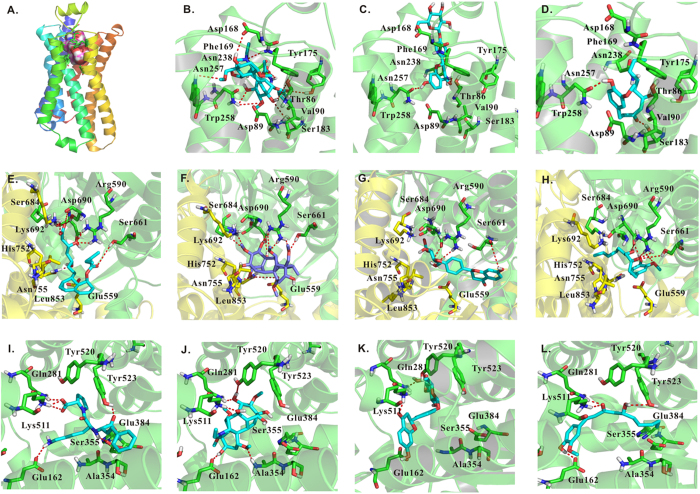
Molecular docking analysis for ADRB1, ACE, and HMGCR with aconitine, liquiritin and 6-gingerol. (**A**) Homology model and the binding pocket for human ADRB1. Detailed interaction mode between (**B**) aconitine/(**C**) liquiritin/(**D**) 6-gingerol and ADRB1. Docking analysis of HMGCR with (**E**) mevastatin, (**F**) aconitine, (**G**) liquiritin and (**H**) 6-gingerol. Docking analysis of ACE with (**I**) lisinopril, (**J**) aconitine, (**K**) liquiritin and (**L**) 6-gingerol.

Since human ADRB1 has no available crystal structure, a homology model for human ADRB1 was built based on the crystal structure of turkey beta-1 adrenergic receptor (PDB Entry: 2Y00) co-crystal structure, which has 73% sequence identity compared with human ADRB1^41^. The pocket was defined according to the key residues in literature report^41^ as shown in Fig. 5A. The detailed docking model for aconitine was shown in Fig. 5B, and it was found that carbonyl and amide in the structure of aconitine can form hydrogen bonds with Val190, Tyr175 and Phe169 respectively. The hydroxyl groups in aconitine also formed hydrogen bonds with residues Thr86, Asn238, Asp89, Asp168 and Asn257, respectively. Hydrophobic interactions were also formed between aconitine and Asp89/Ser183 in ADRB1.

**Figure 6:**
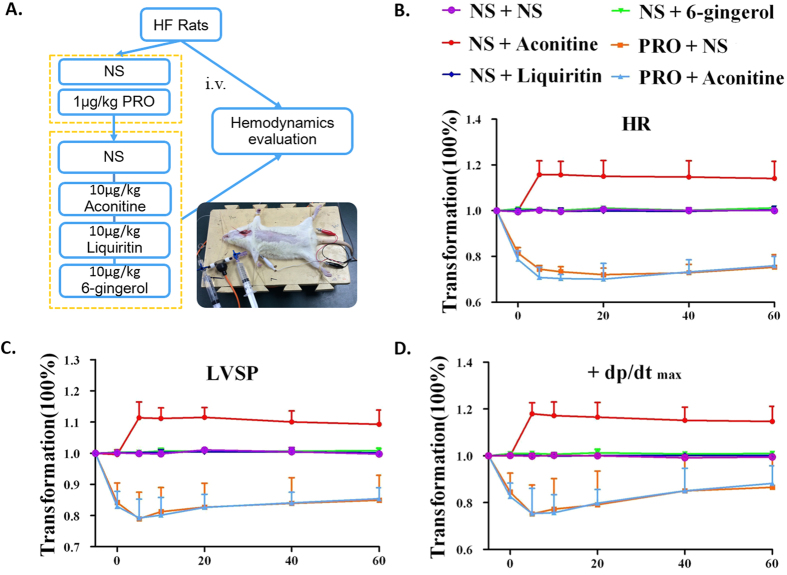
Effects of aconitine, liquiritin, and 6-gingerol alone and combined with propranolol, a beta-1 adrenergic receptor ADRB1 antagonist on heart function. **(A**) Workflow for experiment procedure. (**B**) Effect of drug treatments on heart rate (HR). (**C**) Effect of drug treatments on Left Ventricular Systolic Pressure (LVSP). (**D**) Effect of drug treatments on maximal rate of pressure change (±dp/dt_max_).

By administering simultaneously aconitine and propranolol, it was validated that propranolol, a well-known beta-1 adrenergic antagonist, could block the cardiac inotropic actions induced by aconitine. As shown in Fig. 6, the increase in heart rate (HR, Fig. 6B) and maximum rate of pressure (±dp/dt_max_, Fig. 6D) after administration of aconitine was inhibited by propranolol, indicating aconitine attained the inotropic cardiac function via acting on ADRB1.

However, in the virtual docking analysis of liquiritin (Fig. 5C) and 6-gingerol (Fig. 5D), fewer hydrogen bonds and hydrophobic interactions were formed in the interaction between ADRB1 and liquiritin (one H-Bond) and 6-gingerol (two H-Bonds) than aconitine (eight H-Bonds), indicating a lower binding affinity. This is consistent with the *in vivo* experimental data, which showed no significant change in HR and ±dp/dt_max_ after administration of liquiritin or 6-gingerol (Fig. 6B–D).

### *In Vitro* Binding Studies of HMGCR and ACE with Aconitine, 6-Gingerol and Liquiritin

According to the previous target prediction results, two important targets including HMGCR and ACE might interact with aconitine, 6-gingerol and liquiritin to different degrees. In this section, we performed the protein-binding assay *in vitro* to validate our predictions. Our results showed that aconitine bound to both HMGCR and ACE, while 6-gingerol and liquiritin demonstrated a weak binding affinity, in accordance with our previous virtual docking results.

Multiple compounds in SND targeted HMGCR, the rate-limiting enzyme in the synthesis process of cholesterol. When HMGCR is inhibited, less cholesterol is synthesized, and the liver will uptake more low-density lipoprotein (LDL) from plasma, resulting in lower LDL and cholesterol plasma concentrations. An improved serum lipid profile would enhance circulation and provide a more efficient vessel condition to prevent CVD, such as atherosclerosis, shock and stroke^45^,^46^.

According to the virtual docking results shown in Fig. 5E,F, aconitine (Fig. 5F) and mevastatin (Fig. 5E), a well-known inhibitor in the co-crystal structure of HMGCR^46^, shared a similar binding pose, and both interacted with the same residues in the HMGCR tetramer crystal structure (PDB Entry: 1HW8). Specifically, both mevastatin and aconitine interacted with residues Glu559, Lys692, His752, Asn755 and Leu853 in chain A, and residues Arg590, Ser661, Ser684 and Asp690 in chain B, which contribute to the high binding affinities (Fig. 5F). However, liquiritin (Fig. 5G) and 6-gingerol (Fig. 5H), which had a smaller molecular size, could not occupy the large pocket between the two chains, and mainly interacted with residues in chain B (discussed above), while merely forming a few hydrophobic interactions with residues in chain A. Thus, in accordance with less interaction with chain A, 6-gingerol and liquiritin are predicted to have lower binding affinities compared with mevastatin and aconitine.

Multiple constituents in SND were predicted to act on four targets (ACE, AGTR, AGT and renin) in RAAS^47^. Renin is an enzyme that breaks down angiotensinogen (AGT) into angiotensin I. Angiotensin I is further cleaved in the lung by ACE and transformed into angiotensin II. Angiotensin II acts on angiotensin II receptor type I (AGTR) to produce vasoconstriction and induces the release of aldosterone^48^, which increases re-absorption of sodium and water, thus elevating blood pressure. Moreover, inhibitors for ACE are widely used to treat cardiovascular diseases like hypertension and heart failure. Our plasma biochemistry results show a significant decrease was observed in blood aminopeptidase N (APN) and aldosterone^48^ concentrations was observed in all treatment groups, suggesting that SND might act on RAAS to achieve its function of maintaining blood pressure and improving circulation^47^,^49^.

The interaction modes of aconitine, liquiritin and 6-gingerol with ACE, compared with the interaction with Lisinopril^50^, are shown in Fig. 5I–L. The four compounds all had hydrogen bond acceptors located in a position close to several hydrogen bond donors in Glu162, Gln281 and Lys511, forming hydrogen bonds with these residues at a distance range from 1.5 Å to 3.8 Å. Among them, Lisinopril and aconitine formed more (four H-Bonds) hydrogen bonds than liquiritin (three H-Bonds) and 6-gingerol (two H-Bonds), indicating that the previous two compounds should have relatively higher binding affinities. Additionally, Lisinopril formed two hydrogen bonds with the hydroxyl in Tyr523 and the carbonyl in Ser355, and had a π-π interaction with Tyr523, contributing to its high binding affinity (Ki = 0.27 nM). On the other hand, aconitine and liquiritin have complementary hydrogen bond with Ala354/Tyr520 in a distance of 1.7 Å/2.3 Å, and 2.1 Å/1.9 Å, denoting potential binding affinities lower than Lisinopril but higher than 6-gingerol.

**Figure 7:**
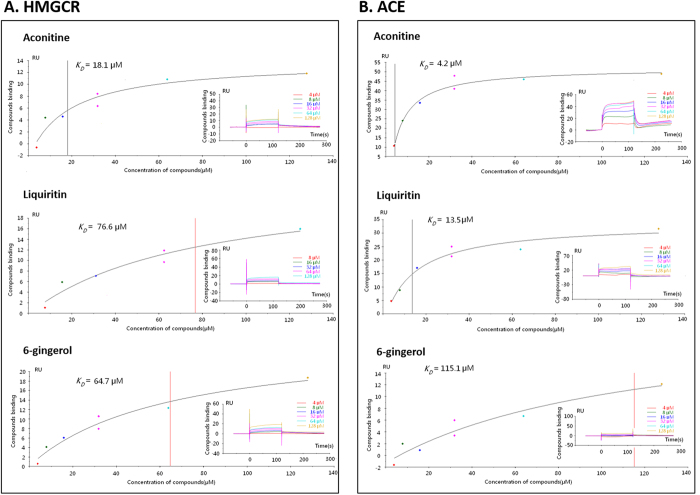
Surface Plasmon Resonance (SPR) analysis of interactions between three major components and two proteins: HMGCR and ACE. (**A**) Binding analysis for HMGCR by SPR assay. (**B**) Binding analysis for ACE by SPR assay.

Here, we further validated whether aconitine, liquiritin and 6-gingerol actually targeted at HMGCR and ACE *in vitro* using Surface Plasmon Resonance (SPR), as shown in Fig. 7. According to SPR results, both HMGCR and ACE bind aconitine. For HMGCR, the binding affinities of aconitine, liquiritin and 6-gingerol were 18.1, 76.6 and 64.7 μM, respectively. For ACE, the binding affinities of aconitine, liquiritin and 6-gingerol were 4.2, 13.5 and 115.1 μM, respectively. These results indicate that aconitine has a higher binding affinity with these two targets than the other two ingredients. These experimental data confirm our computational predictions.

### Synergistic and Antagonistic Effects of Combining Liquiritin and 6-Gingerol with Aconitine and Their Mechanisms

In the formula of SND, *Aconitum carmichaelii* (emperor) plays a dominating role in treating the primary cause of disease^11^. Some researchers claim that aconite and its extract could protect myocardial cells from animal models and patients with heart failure, myocardial infarction, cardiac hypertrophy and other CVDs^51^,^52^. However, aconite is well known as a poisoning herb whose improper use can cause human respiratory paralysis, cardiac arrest and even death^53^.

**Figure 8:**
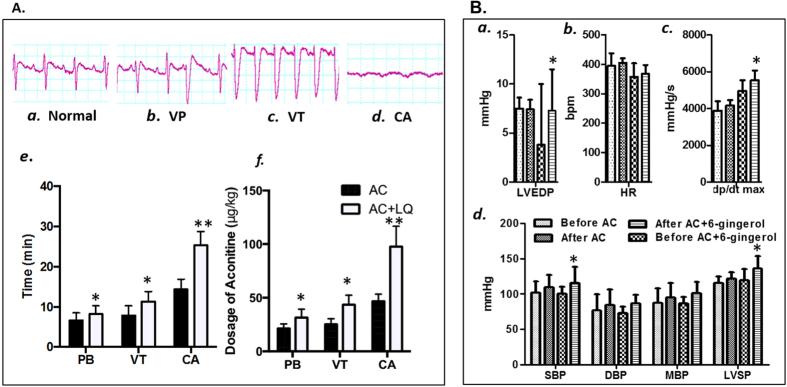
Synergistic and antagonistic effects of components in Sini Decoction. (**A**) Liquiritin (LQ) can alleviate the arrhythmia caused by aconitine (AC). (a–d) The arrhythmia monitored in the ECG after administration of aconitine and liquiritin. (e) Onset time and (f) dosage of arrhythmia after continuous administration of aconitine and liquiritin under PB, VT, and CA. For e and f, solid bars represent AC treatment alone and open bars represent AC + LQ co-treatment. (**B**) 6-gingerol can elevate the cardiac functions of aconitine by hemodynamic indexes. (a) LVEDP, (b) HR, (c) dp/dt_max_, (d) SBP, DBP, MBP and LVSP after administration of aconitine (5 ug/kg) and 6-gingerol (70 ug/kg).) *p < 0.05, **p < 0.01 vs aconitine group. Data shows as mean ± SD.

With the role of “zuo-shi” (adjuvant-courier) in SND formulation, licorice is used to reduce the toxicity of aconite in fighting arrhythmia and performing glucuronide-like detoxification^54^,^55^. Licorice is also a unique “guide drug” in traditional CMF to lead the active constituents in herbs to their target tissues and organs^56^. Liquiritin, our selected representative ingredient of *Glycyrrhiza uralensis*, was validated to alleviate arrhythmia induced by aconitine in this research. The typical electrocardiograms of normal and arrhythmia condition, including premature beats (PB), ventricular tachycardia (VT) and cardiac arrest (CA), were shown in Fig. 8A. The results showed that liquiritin, at a dose of 1 mg/kg, could significantly delay the occurrence of PB, VT and CA induced by aconitine (10 μg/mL at a speed of 0.1 mL/min, i.v.). The calculations of time vs concentration or dosages of aconitine, which could lead to PB, VT and CA, are shown in Fig. 8A, under. It was found that pro-administration of liquiritin resulted in an increase in the dosage of aconitine or longer aconitine treatment in order to induce arrhythmia. These results showed that the liquiritin could ease the arrhythmia caused by aconitine, and thus reduce the toxicity of aconitine.

Some constituents in *Glycyrrhiza uralensis,* especially liquiritin, were predicted to interact with potassium and sodium ion channels, which are drug targets for arrhythmia^57^. It was observed in the above study that administration of liquiritin could alleviate the arrhythmia caused by aconitine in rats. Some researchers have reported that both liquiritin and aconitine could adjust sodium and potassium ion channels, and liquiritin might reduce the cardiac toxicity that caused by aconitine through sodium or potassium channels (Yan Liu, 2008 #73) (Xiaoying Hu, 1996 #72).

Furthermore, it has been previously reported that the *Aconitum carmichaelii*-*Zingiber officinale* pair has been used for more than 2000 years to improve the therapeutic effect of aconitum in curing heart failure^55^,^58^. According to our prediction, 6-gingerol might act on HMGCR to improve the serum lipid profile, and interact with ACE to dilate capillaries in order to improve circulation, thus improving the therapeutic efficacy of *Aconitum carmichaelii*. These pharmacological actions were assessed by the administration of aconitine combined with 6-gingerol measured by hemodynamic indexes. As shown in Fig. 8B, treatment with aconitine resulted in a significant improvement in heart function, as reflected in an increase of LVSP, LVEDP, HR, systolic blood pressure (SBP), diastolic blood pressure (DBP) and ±dp/dt_maz_. Combined with 6-gingerol (aconitine: 6-gingerol = 1:7), the inotropic cardiac effect of aconitine was further enhanced with a significant raise in blood pressure (LVSP, SBP, DBP and MBP) and ±dp/dt _max_. We suggest that aconitine was the main ingredient that achieves the therapeutic effect in SND; 6-gingerol can enhance the cardiac effects of aconitine and attain synergistic actions; liquiritin can ease the arrhythmia resulting from aconitine. These results further validate our predictions.

## Conclusion

In the present study, a domain-specific chemogenomics database for cardiovascular diseases was the first constructed and computational tools were utilized to predict specific targets for multiple constituents in SND. In order to obtain a better understanding of the underlying principle of TCM, we applied the *in silico* systems pharmacology approach to analyze the interaction networks between active constituents and targets. The results of network pharmacology studies, namely the predicted active ingredients and their association with targets, were validated by experimental bioassays.

We predicted, and biological experiments confirmed that aconitine, liquiritin and 6-gingerol are the representative active ingredients of *Aconitum carmichaeli, Zingiber officinale* and *Glycyrrhiza uralensis* respectively in SND. Moreover, our prediction showed that aconitine mainly targets ADRB1 to achieve its cardiac effects, which was further evaluated through co-administration of propranolol (an ADRB blocker). Based on the molecular docking results, we did *in vitro* studies on ingredient-protein binding by SPR, showing that the aconitine and liquiritin can bind to ACE and regulate blood pressure, while aconitine and 6-gingerol target HMGCR.

In addition, our established systems pharmacology method was utilized to investigate synergistic effects and toxicities of components in a complicated herbal formula for the re-evaluation of TCM formula on clinical efficacy. It is known that aconitine might induce arrhythmia at a high dose^53^. We predicted and confirmed that the presence of liquiritin in SND could alleviate the arrhythmia dose-dependently by modulating the sodium ion channel. Meanwhile, when co-administered with 6-gingerol, cardiac effects of aconitine can increase synergistically.

Thus, our results demonstrate the utility of *in silico* pharmacology in predicting the main active components in a complex herbal mixture for a given physiological system. Moreover, using system-specific chemoinformatic tools and network pharmacology, the molecular mechanisms, potential toxicity and synergisms can be evaluated.

## Material and Methods

### Database Construction

*CVDPlatform* was rooted from the established web-interface molecular database prototype CBID (www.cbligand.org) and our recent reports^18^,^22^,^28^,^42^, which was constructed with a MySQL^59^ database and an apache web server, and implemented with in-house cheminformatics tools^28^. Candidate proteins were collected, and their corresponding X-ray crystallographic structures were obtained directly from RCSB Protein Data Bank to build the CVD-specific chemogenomics database (www.cbligand.org/CVD). The homology models of several important CVD targets without crystal structures were constructed, tested and included in the database, such as ADRB1 and TRPV1^30^. The CVD-related pathways, bioassays and references were also obtained from KEGG, DrugBank^60^, PubChem^60^ and CHEMBL^61^.

### High-Throughput Docking (HTDocking)

An online high-throughput docking program was constructed in the *CVDPlatform*, to explore the interaction between the druggable therapeutic targets and chemical compounds. HTDocking is based on the fitness of a small molecule and the protein binding pocket^62^. Briefly, small molecules were docked to the pocket of target proteins related to cardiovascular disease in order to partially avoid the nonspecific binding as reported by Chen YC and co-workers^63^. We also filtered the compounds using PAINS to reduce the effect of non-specific covalent binding between ligand and proteins, since some compounds have groups that can form non-specific covalent binding with many proteins.

We used AutoDock Vina, which offered a multi-facet capability, high-performance rate, and enhanced accuracy, to perform the docking^64^. AutoDock Vina can provide 3–5 predicted binding affinity values (ΔG) from different docking poses for each compound in a binding pocket of a protein. In our HTDocking program, we only considered the proteins with two (or more than two) out of three high binding affinities as potential targets to avoid some false positive situation. We then ranked the potential cardiovascular therapeutic targets according to the docking scores, *pKi* = −log (predicted *Ki*), from each protein structure. Top listed targets with higher docking scores may have higher binding affinities or more chances to interact with our input compounds.

### Compound Library Construction

Based on previous studies on chemical analyses of Sini Decoction^32^, 347 compounds were reported in Sini Decoction, with 196 constituents in *Glycyrrhiza uralensis*, 49 constituents in *Aconitum carmichaelii* and 102 constituents in *Zingiber officinale*. We selected 40 representative compounds with diverse chemical structures, which have been identified and isolated, including 25 constituents in *Aconitum carmichaelii*, 13 constituents in *Glycyrrhiza uralensis* and 2 constituents in *Zingiber officinale* (Table S3).

### Target Prediction and Network Construction

In this study, the molecular docking approach was applied to predict the possible interactions between 40 components from SND and 984 target proteins that integrated into our database. The docking studies were done by our HTDocking program. In order to predict possible targets for synergistic and antagonistic effect and to understand the underlying principle of herbal formulation, we used Cytoscape 3.0.2 to generate, analyze and visualize the graphical network between targets and compounds.

### Animals

All animal experimental protocols were approved by the Administrative Committee of Experimental Animal Care and Use of Second Military Medical University in China (SMMU, Licence No. 2011023), and conformed to the National Institute of Health guidelines on the ethical use of animals. The animal experiments were performed in accordance with the relevant guidelines and national legislation regulations at the Centre of Laboratory Animals of the Second Military Medical University (Shanghai, China). All surgeries were performed under 25% urethane anesthesia and all efforts were made to minimize suffering. Male Sprague-Dawley rats used in this study were supplied by Sino-British Sippr/BKLab Animal Ltd (Shanghai, China).

### HF Rat Models

Fifty SD rats were anesthetized with an intraperitoneal injection of Urethane (1.4 g/kg, i.p.). Their left anterior descending arteries (LAD) were occluded. To prevent infection, rats were given with penicillin (40.000 units) after the operation for 3 days. Fifty animals survived throughout the experiment, including 40 LAD rats and 10 sham rats (without ligation). Forty LAD rats were randomly divided into four groups, 10 in HF model (water), 10 in herbs group (Sini Decoction, 5 g/kg), 10 in components group (three herbs extract, 15 mg/kg) and 10 in compounds group (three active compounds, 1 mg/kg). All drugs were given through oral administration beginning at 4 weeks post-surgery. The drugs were diluted with distilled drinking water and administered orally with a volume of 5 mL/kg body weight once every morning for 4 weeks^65^.

### Echocardiography Assessment

Echocardiography was performed 21 days after surgery according to reported methods. Ten rats from each group were anesthetized by intraperitoneal injection of 100 mg/kg ketamine. After cleaning the rat chest, the left ventricular internal dimension systole (LVIDs), left ventricular internal dimension diastole (LVIDd), left ventricular end-systolic volume (LVESV), left ventricular end-diastolic volume (LVEDV), left ventricular ejection fraction (LVEF) and fractional shortening of left ventricular short axis (LVFS) were measured using a Visual Sonics Vevo770 machine equipped with 23 (or 30) MHz transducers to assess cardiac function. The data calculations were performed using a single blind method^35^,^66^.

### Morphometric Analysis

Myocardial tissues in the left ventricle (LV) of sacrificed rats (approximately 2 mm in thickness) were removed after echocardiography assessment. Samples were fixed in 4% pre-cooled paraformaldehyde for 72 h and embedded in paraffin for histological studies. Paraffin-embedded tissues were sectioned into slices of about 5 mm thicknesses. Masson’s trichromatic staining was performed to assess myocardial fibrosis. Images were visualized under an optical microscope at x400 magnification^67^.

### Validation of Aconitine with Beta-adrenergic receptor *in vivo*

SD rats were anesthetized with an intraperitoneal injection of urethane (1.4 g/kg, i.p.). The first polyethylene catheter, connected to a pressure transducer, which was equipped with a polygraph, was inserted into the right carotid artery and then advanced into the left ventricle cavity to record LVSP, LVEDP and HR. The second polyethylene catheter was inserted into the femoral vein for drug administration. The HR, LVSP and ±dp/dt_max_ were analyzed by Lab chart software.

### Surface Plasmon Resonance (SPR) analysis of HMGCR and ACE

SPR kinetics experiments of HMGCR and ACE with 3 ingredients were carried out in a Biacore T200 system (GE Healthcare, Sweden). HMGCR and ACE were diluted in sodium acetate pH 4.5 (GE Healthcare) and immobilized by the amine coupling method on a CM5 sensor chip according to the manufacturer’s protocol (GE Healthcare). Flow cells 2 through 4 were immobilized with a ligand, and flow cell 1 was equally treated but without protein as a control. Aconitine, liquiritin and 6-gingerol were diluted in HBS-EP+ running buffer at concentrations ranging from 4 μM to 128 μM, generally with a series of six 2-fold escalations to a maximum concentration about 10-fold higher than the *K*_*d*_, with duplicate middle concentration running at the end of each run to confirm the stability of the sensor surface. Analytes were injected at a flow rate of 30 μL/min. The association and dissociation times were 60 seconds and 300 seconds, respectively. The affinity fitting was carried out with Biacore T200 evaluation software by a global fitting using the steady state affinity model, and the kinetics data *K*_*d*_ was calculated^68^,^69^,^70^,^71^.

### Evaluation of Synergism and Antagonism and Attenuation of Liquiritin and 6-Gingerol on Aconitine

Male SD rats weighing 280–300 g were equally divided into two groups randomly: A (Aconitine) and B (Aconitine + Liquiritin). SD rats were anesthetized with an intraperitoneal injection of urethane (1.4 g/kg, i.p). The rats were injected intravenously with normal saline in group A and with 4 mg/kg Liquiritin in group B. After 5 min, 10 μg/ml aconitine was injected into the rats at a constant rate. The cardiac function was monitored on a PowerLab 8/35 (AD instrument, Australia), and the rats were connected to PowerLab through three electrocardiograph electrodes. The electrocardiogram was recorded to show the appearance time of PB, VT and CA, and to calculate the dosage of aconitine, which could lead to PB, VT and CA.

SD rats were anesthetized with an intraperitoneal injection of Urethane (1.4 g/kg, i.p.). The first polyethylene catheter was inserted into the right carotid artery and then advanced into the left ventricle cavity to record left ventricular systolic (LVSP) and heart rate (HR). The second polyethylene catheter was inserted into the lower abdominal aorta through the left femoral artery to record SBP and DBP. The third polyethylene catheter was inserted into a femoral vein for drug administration. The HR, LVSP, LVEDP, SBP, DBP, MBP, and maximal rate of pressure development (+dp/dt) and decline (−dp/dt) were analyzed by Lab chart software.

## Additional Information

**How to cite this article**: Zhang, H. *et al.* Cardiovascular Disease Chemogenomics Knowledgebase-Guided Target Identification and Drug Synergy Mechanism Study of an Herbal Formula. *Sci. Rep.* **6**, 33963; doi: 10.1038/srep33963 (2016).

## Supplementary Material

Supplementary Information
