# Supplementary material for: Cardiovascular Disease Chemogenomics Knowledgebase-guided Target Identification and Drug Synergy Mechanism Study of an Herbal Formula
Source: Sci Rep. 2016 Sep 28;6:33963. doi: 10.1038/srep33963 (PMC5039409; doi:10.1038/srep33963)
Supplement: Supplementary Information [file srep33963-s1.doc]

**Supplement Information**

**Cardiovascular Disease Chemogenomics Knowledgebase-Guided Target Identification and Drug Synergy Mechanism Study of an Herbal Formula**

Hai Zhang^1#^, Shifan Ma^2#^, Zhiwei Feng^2#^, Dongyao Wang^1#^, Chengjian Li^1^, Yan Cao^1^, Xiaofei Chen^1^, Aijun Liu^1^, Zhenyu Zhu^1^, Junping Zhang^1^, Guoqing Zhang^1^, Yifeng Chai^1*^, Lirong Wang^2*^, and Xiang-Qun Xie^2*^

^1^College of pharmacy, Second Military Medical University; Department of Pharmacy, Third Affiliated Hospital of Second Military Medical University, Shanghai 200433, China;

^2^Department of Pharmaceutical Sciences and Computational Chemical Genomics Screening Center, School of Pharmacy; National Center of Excellence for Computational Drug Abuse Research; Drug Discovery Institute; Departments of Computational Biology and Structural Biology, School of Medicine, University of Pittsburgh, Pittsburgh, Pennsylvania 15260, United States.

^#^Hai Zhang, Shifan Ma, Zhiwei Feng and Dongyao Wang contributed equally to this work.

***Corresponding Author: Xiang-Qun (Sean) Xie, MBA, Ph.D, Yifeng Chai, PhD, Lirong Wang, PhD:**

Xiang-Qun Xie, PhD and EMBA, Department of Pharmaceutical Sciences and Computational Chemical Genomics Screening Center, School of Pharmacy, University of Pittsburgh, Pittsburgh, Pennsylvania 15260, United States. Email: [xix15@pitt.edu](mailto:xix15@pitt.edu)

Yifeng Chai, PhD, College of pharmacy, Second Military Medical University, Shanghai 200433, China; Email: [yfchai@smmu.edu.cn](mailto:yfchai@smmu.edu.cn)

Lirong Wang, PhD, Department of Pharmaceutical Sciences and Computational Chemical Genomics Screening Center, School of Pharmacy, University of Pittsburgh, Pittsburgh, Pennsylvania 15260, United States. Email: [liw30@pitt.edu](mailto:liw30@pitt.edu)


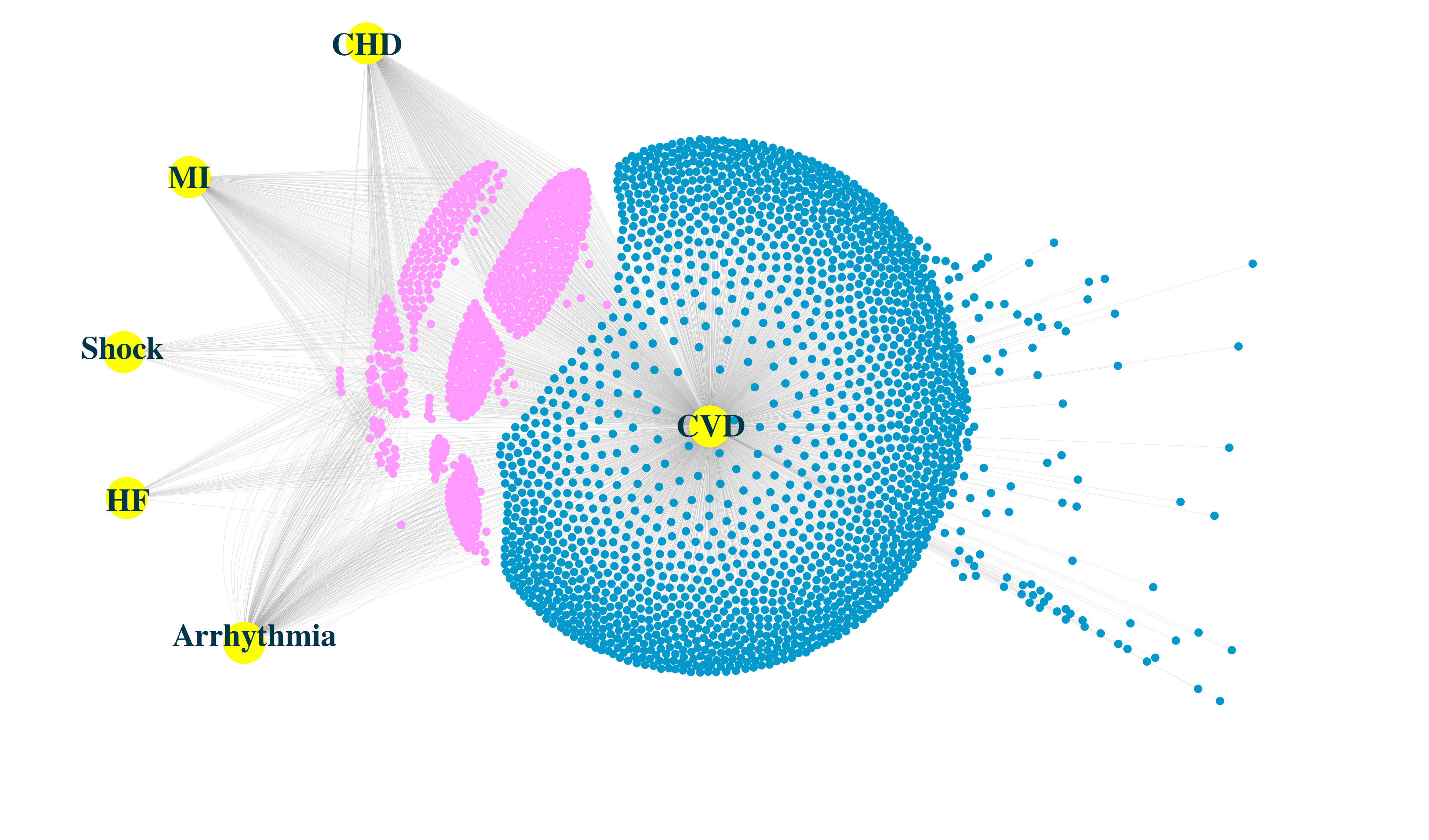


Figure S1. Targets for SND Related Indications and Other Cardiovascular Diseases

Targets in pathological pathways of SND indications, including coronary heart disease, myocardial infarction, heart failure, shock, and arrhythmia, represent by pink nodes; the other targets related with the etiology for other cardiovascular diseases are in blue; the yellow large nodes stand different types of diseases.

##
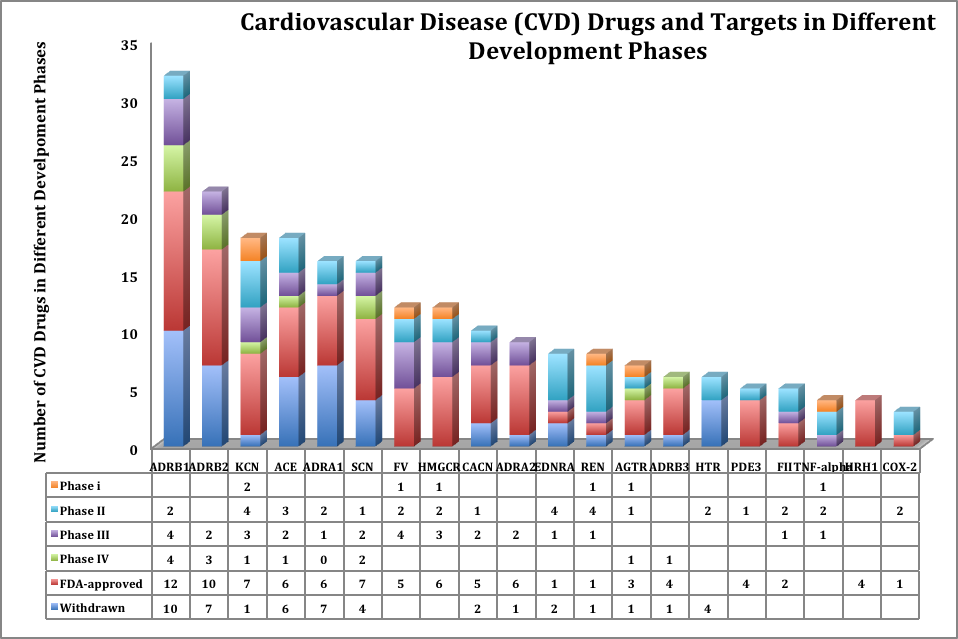


Figure S2. Drugs and Their Targets in Different Development Phases

These approved and clinical trial CVD drugs were classified by different phases, distinguished by distinct colors. The red and blue columns indicate the approved and discontinued CVD drugs, respectively. The orange, light blue, purple and green lines denote clinical trial drugs in phases I, II, II and IV respectively.


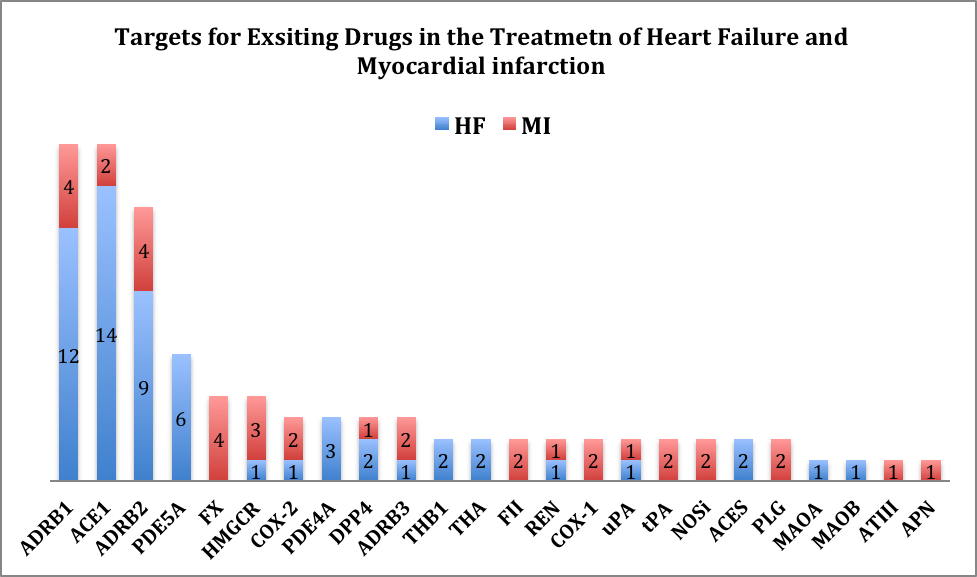


Number of drugs for the target

Figure S3. Therapeutic Targets for MI and HF ranked by the number of drugs

These approved and clinical trial drugs were classified by treatment for MI and HF, distinguished by distinct colors. The red columns indicate MI drugs; the blue columns denote HF drugs. The targets are ranked by the total number of drugs for the treatment of two diseases.


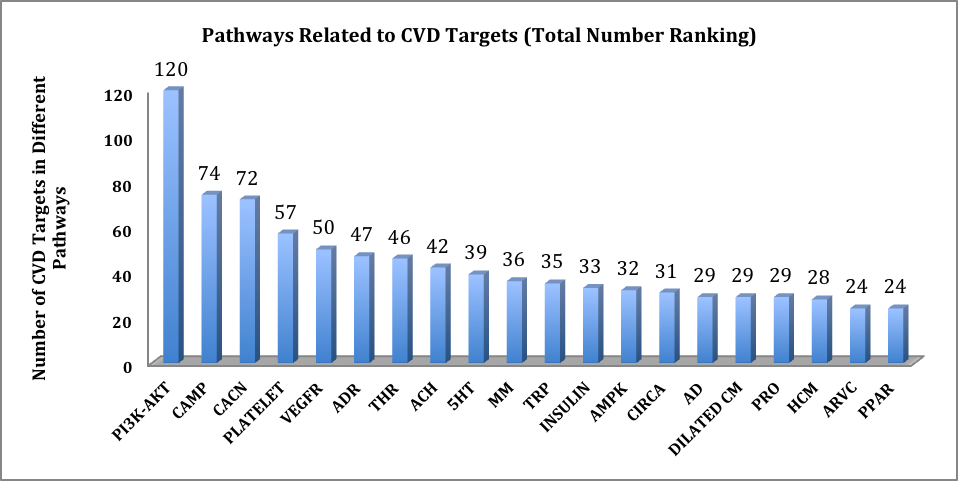


**A**.


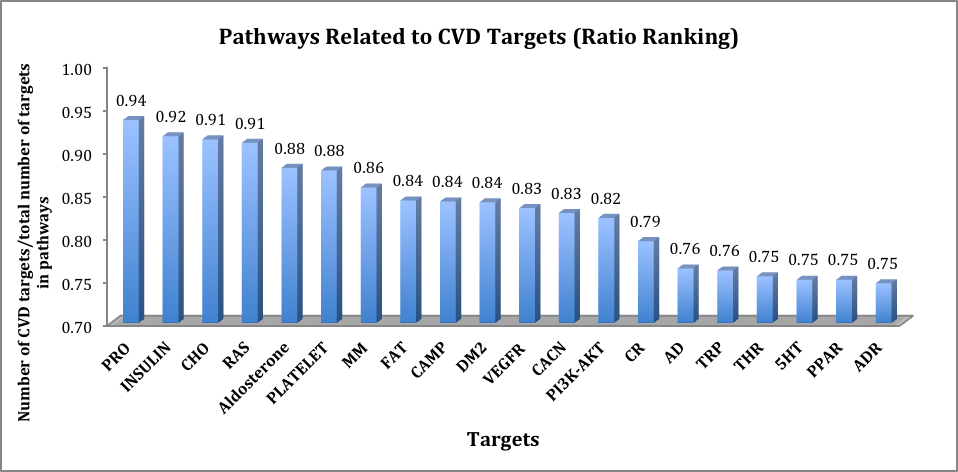


**B**.

Figure S4. CVD Related Pathways in *CVDPlatform*

The CVD related pathways were plotted according to the number of CVD targets involved. 1 The top 20 pathways with more than 20 CVD targets involved were listed. (B) The number of CVD drugs in a pathway was divided by the total number of the targets in the pathway.

Table S1. Comparison of the Experimental Data and the Predicted Results

| Target | Drug | Docking Score | Exp  pKi | Exp  Ki(nM) | Reference/ PubChem ID |
| --- | --- | --- | --- | --- | --- |
| Coagulation Factor Xa | Apixaban | 8.75 | 9.09 | 0.8 | J. Med. Chem., (2007) 50:22:5339 |
| Coagulation Factor II | Argatroban | 8.04 | 8.39 | 4 | J. Med. Chem., (2003) 46:17:3612 |
| Coagulation Factor III | Argatroban | 7.27 | 7.40 | 39 | J. Med. Chem., (2003) 46:17:3612 |
| 3-Hydroxy-3-Methylglutaryl-Coenzyme A Reductase | Lovastatin | 7.52 | 8.22 | 6 | AID83293 |
| Coagulation Factor Xa | Argatroban | 6.26 | 5.27 | 5300 | AID766528 |
| Angiotensin-Converting Enzyme | Captopril | 9.12 | 8.76 | 1.7 | AID37801 |
| Renin | Captopril | 8.98 | 8.76 | 1.7 | AID198995 |
| Coagulation Factor II | Apixaban | 6.54 | 5.50 | 3100 | AID302354 |
| Cholinesterase | Lovastatin | 7.22 | 5.95 | 1100 | AID1093611 |
| 3-Hydroxy-3-Methylglutaryl-Coenzyme A Reductase | Fluvastatin | 11.83 | IC50=28nM | | Bioorg. Med. Chem. Lett., (2005) 15:4:1027 |
| Renin | Aliskiren | 6.58 | IC50=0.5nM | | AID371780 |
| 3-Hydroxy-3-Methylglutaryl-Coenzyme A Reductase | Atorvastatin | 10.57 | IC50=0.227nM | | AID625271 |
| 3-Hydroxy-3-Methylglutaryl-Coenzyme A Reductase | Pravastatin | 8.37 | IC50-5.59nM | | AID625271 |
| Angiotensin-Converting Enzyme | Trandolapril | 8.53 | IC50=0.93nM | | AID39767 |
| Urokinase-Type Plasminogen Activator Precursor | Fluvastatin | 7.69 | Potency=56.2nM | | AID540303 |
| Glucocorticoid Receptor | Fluvastatin | 7.32 | Potency=264.8nM | | AID720692 |

Table S2. The Validated Interaction between SND Components and Predicted Targets

| **Compound Name** | **Target Abberviation** | **Target Name** | **Assay** | **Value** | **Reference** |
| --- | --- | --- | --- | --- | --- |
| **6-gingerol** | AChE | [Acetylcholinesterase](http://www.ncbi.nlm.nih.gov/protein?term=Acetylcholinesterase) | Ki | 0.0106 | 5 |
| **6-gingerol** | TRPV1 | Transient receptor potential cation channel subfamily A member 1 | Efficacy | 0.114 | 6 |
| **6-gingerol** | LTAH4 | [leukotriene A4 hydrolase](http://cancerres.aacrjournals.org/content/69/13/5584.short) | Efficacy | 0.114 | 1 |
| **aconitine** | [CHRNA7](http://www.uniprot.org/uniprot/P36544) | Neuronal acetylcholine receptor protein alpha-7 subunit | Ki | 2.12nM | 7 8 |
| **glycyrrhetinic acid** | PPARG | Peroxisome proliferator-activated receptor gamma | Activity | Active | 9 |
| **glycyrrhetinic acid** | HSD-11β | [11-beta-hydroxysteroid dehydrogenase 2](http://www.ncbi.nlm.nih.gov/protein?term=11-beta-hydroxysteroid dehydrogenase 2) | IC50 | 1nM | 10 |
| **glycyrrhetinic acid** | AChE | [Acetylcholinesterase](http://www.ncbi.nlm.nih.gov/protein?term=Acetylcholinesterase) | INH | 0.0312 | 11 |
| **liquiritin** | [ADRB2](http://www.uniprot.org/uniprot/P07550) | Beta-2 adrenergic receptor | Potency | 6.5131uM |  |
| **glycyrrhetinic acid** | AKR1B10 | Aldo-keto reductase family 1 member B10 | IC50 | =4.9uM | 12 |
| **glycyrrhetinic acid** | POLL | [DNA polymerase kappa](http://www.ncbi.nlm.nih.gov/protein?term=DNA polymerase kappa) |  |  | 13 |
| **Aconitine** |  | tetrodotoxin-sensitive, voltage-dependent sodium channels |  |  | 14 |
| **Glycyrin** | GCR | Glucocorticoids receptor | IC50 | 2.6uM | 15 |
| **Higenamine** | ADRB1 | Beta-1 adrenergic receptor |  |  | 16 |

Table S3: Constituents and their chemical structures in SND

|  | **Name** | **Structure** |  | **Name** | **Structure** |
| --- | --- | --- | --- | --- | --- |
| **1** | Aconitine |  | 20 | Benzoylnapelline |  |
| **2** | Hypaconitine |  | 21 | Isobenzoylnapelline |  |
| **3** | Mesaconitine |  | 22 | Higenamine |  |
| **4** | Deoxyaconitine |  | 23 | Coryneine |  |
| **5** | Pyroaconitine |  | 24 | Isotalatizidine |  |
| **6** | Pyrohypaconitine |  | 25 | Salsolinol |  |
| **7** | Pyromesaconitine | Table 1 continued | 26 | Glycyrrhizic Acid |  |
| **8** | Benzoylaconine |  | 27 | Glycyrrhizin |  |
| **9** | Benzoylhypaconine |  | 28 | Isoliquiritin |  |
| **10** | Benzoylmesaconine |  | 29 | Liquiritigenin |  |
| **11** | Benzoyldeoxyaconine |  | 30 | Glycyamarin |  |
| **12** | Aconine |  | 31 | Isoliquiritigenin |  |
| **13** | Hypaconine |  | 32 | Glycyrrhetinic Acid |  |
| **14** | Mesaconine |  | 33 | Glycyrol |  |
| **15** | Neoline |  | 34 | Licoricidin |  |
| **16** | Talatisamine | Table 1 continued | 35 | Iso-Glycyrol |  |
| **17** | Fuziline |  | 36 | Glycyrrhetic Acid |  |
| **18** | 14-Acetyl-Talatisamine |  | 37 | Liquiritin |  |
| **19** | Talatizidine |  | 38 | Glycyrin |  |
| **39** | 6-Gingerol |  | 40 | 8-gingerol |  |

1-25 is form Aconitum carmichaelii, 26-38 is form Glycyrrhiza uralensis, 39 and 40 is from Zingiber officinale

**Reference**
